# Advancing a Framework to Enable Characterization and Evaluation of Data Streams Useful for Biosurveillance

**DOI:** 10.1371/journal.pone.0083730

**Published:** 2014-01-02

**Authors:** Kristen J. Margevicius, Nicholas Generous, Kirsten J. Taylor-McCabe, Mac Brown, W. Brent Daniel, Lauren Castro, Andrea Hengartner, Alina Deshpande

**Affiliations:** 1 Defense Systems and Analysis Division, Los Alamos National Laboratory, Los Alamos, New Mexico, United States of America; 2 Biosciences Division, Los Alamos National Laboratory, Los Alamos, New Mexico, United States of America; Inserm & Universite Pierre et Marie Curie, France

## Abstract

In recent years, biosurveillance has become the buzzword under which a diverse set of ideas and activities regarding detecting and mitigating biological threats are incorporated depending on context and perspective. Increasingly, biosurveillance practice has become global and interdisciplinary, requiring information and resources across public health, One Health, and biothreat domains. Even within the scope of infectious disease surveillance, multiple systems, data sources, and tools are used with varying and often unknown effectiveness. Evaluating the impact and utility of state-of-the-art biosurveillance is, in part, confounded by the complexity of the systems and the information derived from them. We present a novel approach conceptualizing biosurveillance from the perspective of the fundamental data streams that have been or could be used for biosurveillance and to systematically structure a framework that can be universally applicable for use in evaluating and understanding a wide range of biosurveillance activities. Moreover, the Biosurveillance Data Stream Framework and associated definitions are proposed as a starting point to facilitate the development of a standardized lexicon for biosurveillance and characterization of currently used and newly emerging data streams. Criteria for building the data stream framework were developed from an examination of the literature, analysis of information on operational infectious disease biosurveillance systems, and consultation with experts in the area of biosurveillance. To demonstrate utility, the framework and definitions were used as the basis for a schema of a relational database for biosurveillance resources and in the development and use of a decision support tool for data stream evaluation.

## Introduction

Detecting disease outbreaks, surveillance of mass events[1], reporting public health emergencies of international concern (PHEIC) to comply with the International Health Regulations (IHR 2005)[2], [3], and monitoring and predicting the emergence and re-emergence of infectious disease[4] all now fall under the vast biosurveillance umbrella. Biosurveillance systems have been implemented and are being developed to meet these demands, among others, for the gathering and analysis of information that can lead to actionable results at the local, state, national, and global levels for animal, plant, and human populations[5]–[16].

This array of systems spans boundaries between public health surveillance and bioterrorism surveillance, between surveillance centered on health threats and surveillance centered on health protection and monitoring. Under the rubric of biosurveillance, traditional demarcations are increasingly fading among public health, animal health, ecological health, and biosecurity and bioterrorism defense surveillance capabilities. The 2012 National Strategy for Biosurveillance underscores this ‘big umbrella’ approach to biosurveillance by calling for a well-integrated national biosurveillance enterprise to “detect, track, investigate, and navigate incidents affecting human, animal, and plant health, thereby better protecting the safety, well-being, and security of the American people”[17].

There have been repeated calls for consensus definitions and a comprehensive strategy to discuss, compare, and evaluate the dynamically changing and expanding arena of biosurveillance within multiple disciplines[18]–[21]. Definitions and terminology specific to, or applied to, biosurveillance come from disparate fields and have resulted in an *ad hoc* lexicon of terms that are not consistently defined, the meaning of biosurveillance being just one example[22]. When confronted with the need to evaluate the benefits and effectiveness of biosurveillance systems, various definitions, categories, and frameworks have been applied[9], [23]–[25], frequently using the oft-cited CDC guidelines for evaluating public health surveillance systems[26].

A challenge in the field of infectious disease surveillance is the meaningful use of complex, disparate, and information-rich data sets to facilitate achievement of various surveillance goals. Determining *what* data is available to use, identifying the *most useful* data streams, and *using* the data streams to achieve a surveillance goal, all present unique requirements. While numerous local, national, and global disease surveillance systems have been implemented to meet the demands of monitoring, detecting, and reporting disease outbreaks and prevalence, varying surveillance goals and geographic reach have led to multiple and disparate systems, each using unique combinations of data sources to meet surveillance criteria. Rather than a systematic approach, data sources have been selected primarily on what is available quickly and easily. As a result, the impact that big data, such as genomics, metagenomics, climate data, satellite imagery, social networks, etc., may have in biosurveillance is unclear due to the challenges described above. In this context, it is necessary to have a defined framework to enable characterization of this data and to facilitate understanding its utility.

Within the context of biosurveillance systems, data streams are inextricably linked to the system in which they are deployed and are usually evaluated in the context of system capabilities (such as timeliness and sensitivity)[23], [27], [28]. As part of a larger project to provide a systematic evaluation of data streams for integrated global biosurveillance, we required concrete terminology to enable the cataloging, characterization, and classification of diverse data sources and systems that exist or are being developed for use in infectious disease biosurveillance.

Evaluation of data streams as opposed to evaluation of surveillance systems requires a characteristic set of attributes and metrics that are related to, but are not the same as, system attributes and metrics. Because biosurveillance systems can include one or many types of data streams, a data stream-centric framework can also be used in biosurveillance system evaluation and discourse if the framework is based on cogent classification schemes and definitions.

The Biosurveillance Data Stream Framework and biosurveillance definitions described in this paper are presented as a meaningful step towards building a biosurveillance lexicon. Moreover, we submit that this framework and lexicon would support analysis and evaluation of developing systems and data streams through a comprehensive and measurable approach serving as a platform for relative comparisons that can drive collaboration within and beyond the biosurveillance community.

## Methods

To develop working definitions and a categorization framework for data streams that have been or could be useful in a biosurveillance system, relevant information was collected in three ways: through the survey of a subject matter expert (SME) panel, a literature search, and a survey of current operational biosurveillance systems (local, national, and global).

An SME panel was established representing experts involved in animal, plant, and human infectious disease biosurveillance activities or development from U.S. federal government agencies, national laboratories, and academic institutions (Table 1). The panel consisted primarily of research-based individuals (>75%), although individuals working in operational biosurveillance were also included. Details of the development of this panel can be found in Deshpande et al[29]. The primary purpose of the panel was to elicit expert opinion, through a multipart 10-question survey (Table 2), regarding the current state of understanding about global disease biosurveillance, integrated biosurveillance, biosurveillance goals, traditional and non-traditional data streams used or useful for infectious disease biosurveillance, biosurveillance system metrics, and priority diseases for biosurveillance. Twenty-eight survey responses were received; surveys were individual responses except for three, which were joint efforts from SMEs within the same institution.

**Table 1:** Subject Matter Expert Panel Representation, by Agency.

U.S. Southern Command (DoD/SOCOM)
Office of the Assistant to the Secretary of Defense for Chemical and Biological Defense (OASD, NCB/CB)
Armed Forces Health Surveillance Center (AFHSC)
United States Public Health Service (USPHS)
Centers for Disease Control and Protection (CDC)
Los Alamos National Laboratory (LANL)
Oak Ridge National Laboratory (ORNL)
Pacific Northwest National Laboratory (PNNL)
Global Viral Forecasting (GVFInc)
George Washington University (GWU)
Harvard University
Johns Hopkins University, Applied Physics Lab (JHU/APL)
University of Missouri (MU)
Texas A&M University
University of Minnesota (UMN)
Western University of Health Sciences, College of Veterinary Medicine (Western U)
Oklahoma State University (OSU)
Animal and Plant Health Inspection Service (USDA, APHIS)
National Animal Health Laboratory Network (USDA, NAHLN)
Animal and Plant Health Inspection Service, Center for Plant Health Science and Technology (USDA, APHIS, CPHST)

**Table 2:** Survey Questions Answered by Subject Matter Expert Panel.

1) What is your brief definition of the following terms: biosurveillance, global biosurveillance, integrated biosurveillance, data stream, data stream integration, and non-traditional data stream?
2) What are the primary goals of global biosurveillance?
3) Do you think a single integrated global biosurveillance system can fulfill all goals of surveillance? Please elaborate why you do or do not think so.
4) How would you evaluate the utility of a data stream to be used in global biosurveillance? Can you identify a set of metrics (e.g. time to disease detection, ease of accessibility, cost, sustainability, etc.)?
5) Can you rank the metrics in order of importance?
6) Can you provide examples of what you consider useful non-traditional data streams?
7) What in your opinion would be the 10 most important diseases that we could use to evaluate data streams for biosurveillance?
8) What gaps do you see in current biosurveillance systems/strategies?
9) What current technologies do you think are most important to a global biosurveillance system?
10) What near-future technologies do you think will have greatest utility to a global biosurveillance system?

Only contact and affiliation information was collected about the individual SME responding to the questionnaire, and the survey was strictly a means to record expert opinion. Therefore, this survey did not involve human subjects research, and institutional review of the survey was deemed unnecessary (Common Rule (45 CFR 46), LANL Human Subjects Research Review Board).

A literature search was conducted to assess infectious disease biosurveillance terminology and types of data streams used (past, current, or considered for future use) in infectious disease biosurveillance. The intent of the literature search was not to put together a review, but rather to find what kinds and types of data streams have been used in or considered for use by biosurveillance systems to inform our characterization framework. Multiple sources were consulted including book chapters, conference proceedings, peer-reviewed literature, and government reports found through traditional database searching (Web of Knowledge, Google Scholar), references of references, and personal contacts. More than 750 articles were examined for relevance, or uniqueness of possible data streams for biosurveillance. As data streams were identified common characteristics and attributes were cataloged. As the framework developed, the collected data streams were then binned back into the framework classification which informed the development of the data stream categories first by suggesting what the categories should be and second by validating that any data stream was indeed classifiable. Similarly, if a new or novel data stream was found that couldn't be readily binned, the framework was refined, making this process iterative. A list of references that were used in the data stream characterization and the subsequent binning of the data streams from these references into the associated data stream categories is found in the Table S1.

For the scope of our project, a survey of operational infectious disease biosurveillance systems was confined to systems that conducted electronic surveillance using at least one data stream and that performed analysis to report actionable results. Systems were identified through web searches, through a literature search (as described above), and through information provided by the above SME panel or others in the biosurveillance community. Additionally, several past efforts to collect information on biosurveillance systems were also consulted[30], [31]. To better catalog, annotate, and assess biosurveillance systems and resources, a searchable relational database was developed. This effort has continued and is now being advanced at LANL through the creation of the web-hosted Biosurveillance Resource Directory (BRD). The BRD is currently being tested and vetted for scope, open-source functionality, and sustainability as described in detail elsewhere [29]. For each system in the directory, data streams used by the system were recorded and classified in accordance with the evolving data stream framework. Additionally, detailed information about each system and associated data streams was collected and captured in the BRD including specific information regarding how the data was collected, the geographic and population domain/range of the system, the scope of diseases covered by the system, and pertinent documentation (research articles or fact sheets) associated with the system. The systems included in the survey relevant to this paper were current as of October 2012.

## Results and Discussion

### Framework Development

**Figure 1:**
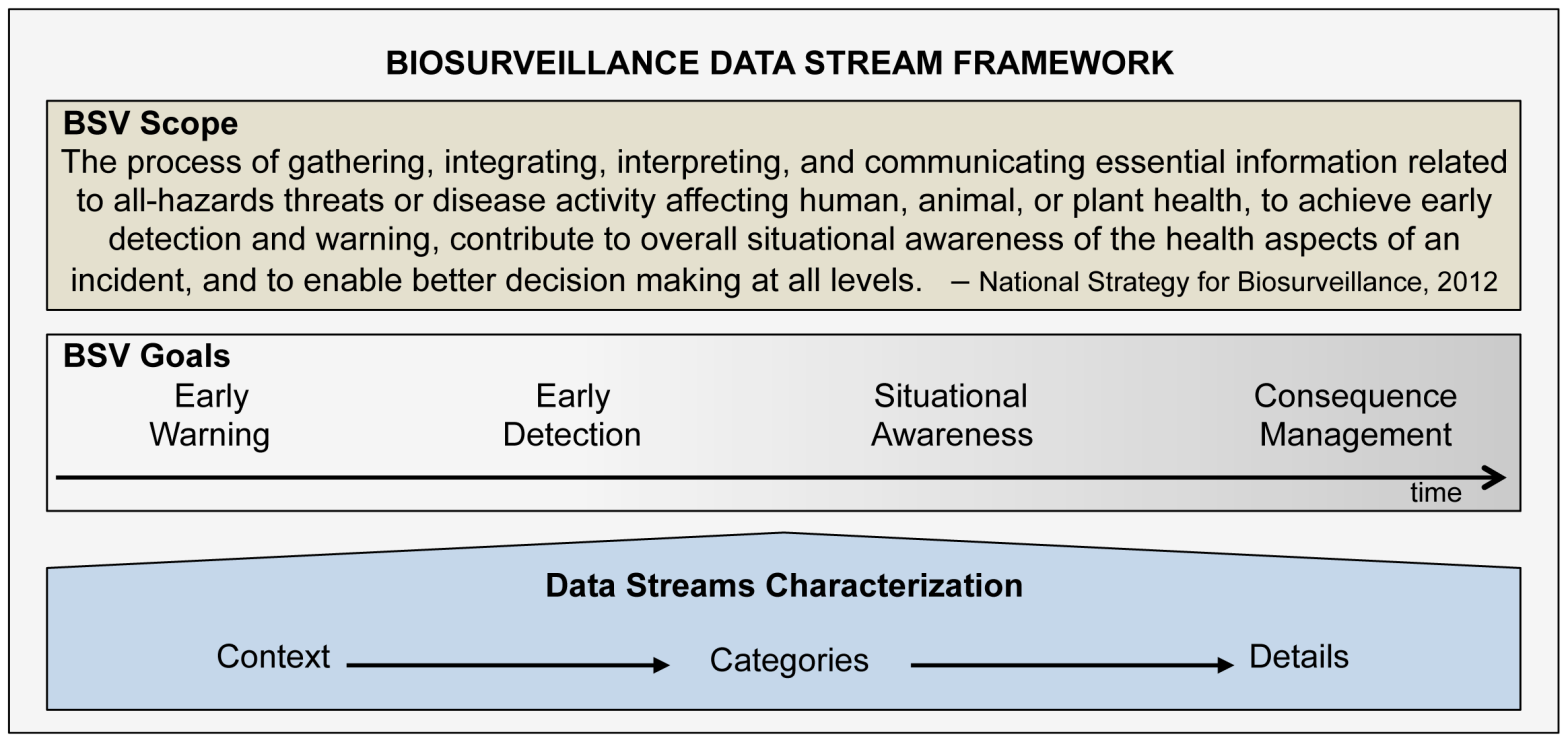
Overview of the Biosurveillance Data Stream Framework.

The focus of our work was to develop a universally applicable framework to categorize any data stream useful for biosurveillance with the intent that the categorization scheme could be used in evaluation and classification. This required a concrete analysis of the scope of biosurveillance, the context in which the data streams would be used (biosurveillance goals), and construction of a data stream characterization method general enough to be useful for comparing data streams, but retaining sufficient detail regarding data stream attributes. These key elements of the developed framework are shown in Figure 1.

#### Biosurveillance Definition and Scope

Biosurveillance has undergone a metamorphosis. Shortly after the anthrax letter attacks in 2001, biosurveillance had come to mean early detection of a bioterrorist threat using computational data algorithms to find patterns and anomalies in electronic health records that could lead to an alert[32], [33]. Biosurveillance was considered a novel, specific, tool:

“We need to pay attention constantly, always on the lookout for any clues that killer bugs have been released in case the next bioterrorist unleashes an attack surreptitiously, instead of with threatening letters. The good news is that computers can help us do this — if we let them. *The technique is called biosurveillance.* By monitoring medical records, smart systems can sift through data and look for connections that humans would probably miss.”[34]

Public health surveillance, biosurveillance's older big sister, has had a much longer history, with modern U.S. public health surveillance definitions typically cited beginning with Langmuir[35] and coming primarily from the CDC.

As a means to understand the scope of biosurveillance and public health surveillance we deconstructed biosurveillance and public health surveillance definitions into what we have found to be three distinguishing components:

**Process**: how surveillance is done; connected to systems and methods of surveillance

**Knowledge**: the information that comes from surveillance - *e.g.,* data streams

**Purpose**: the reasons or intent for the surveillance - *e.g.,* goals.

These components are reflected in both public health surveillance and biosurveillance definitions that have varied over time (Tables 3 and 4). The scope of public health surveillance, as reflected in accepted definitions, has shown that there is not just one kind of public health surveillance, rather public health surveillance encompasses a broad range of data and surveillance methodologies. Table 3 indicates that the process and knowledge has remained relatively constant in our understanding of public health surveillance, but the purpose has had less consensus, especially regarding public health surveillance breadth.

**Table 3:** Public Health Surveillance Definitions.

	Public Health Surveillance: CDC Definitions				
	1988[56]	1992[35], 2012[57]	2001[26]	2011[58]	2008, 2012[37] **
**Process**	The ongoing systematic collection, analysis, and interpretation	The ongoing, systematic collection, analysis, and interpretation	The ongoing, systematic collection, analysis, interpretation, and dissemination	The ongoing, systematic collection, analysis, and interpretation	The systematic, ongoing collection, management, analysis, and interpretation
**Knowledge**	Of health data	Of health data essential to the planning, implementation and evaluation of public health practice	Of data regarding a health-related event	Of health-related data	Of data
**Purpose**	Closely integrated with the timely dissemination of these data both to those providing the data, *and to those who can apply the data to control and prevention programs*	Closely integrated with the dissemination of these data to those who need to know, *and linked to prevention and control* *	For use in public health action, *to reduce morbidity and mortality, and to improve health*	With the *a priori* purpose of preventing or controlling disease or injury, *or of identifying unusual events of public health importance*, followed by the dissemination and use of information, *for public health action*	Followed by the dissemination of these data to public health programs, *to stimulate public health action*

The complete definition in Thacker and Berkelman's 1992 book chapter is “the final link of the surveillance chain is the application of these data to prevention and control. A surveillance system includes a functional capacity for data collection, analysis, and dissemination linked to public health programs”.

Definition that Thacker et al. (2012) cite from the 2008 Dictionary of Epidemiology, 5^th^ ed.

**Table 4:** Biosurveillance Definitions.

Biosurveillance						
	“Technologies for Distributed Defense” 2002[59]	Handbook of Biosurveillance 2006[60]	National Association of County and City Health Officials (NACHHO) 2006[61], 2013[62]	Homeland Security Presidential Directive 2007[36]	National Biosurveillance Integration Center, DHS 2012[42]	National Strategy for Biosurveillance, White House 2012[17]
**Process**	Observing the states of health of a given population by the collection, analysis and correlation	Process that systematically collects and analyzes	Automated monitoring	The process of active data-gathering with appropriate analysis and interpretation	The science and practice of managing	The process of gathering, integrating, interpreting, and communicating
**Knowledge**	Of information derived from a variety of data sources … essentially any and all sources of data	Data	Of existing health data sources	Of biosphere data that might relate to disease activity and threats to human or animal health – whether infectious, toxic, metabolic, or otherwise, and regardless of intentional or natural origin –	Human, animal, plant, food, and environmental health-related data and information	Essential information related to all-hazards threats or disease activity affecting human, animal, or plant health
**Purpose**	That may inform the development of a “disease signature” that marks the presence of disease within a population	For the purpose of detecting cases of disease, outbreaks of disease, and environmental conditions that predispose to disease	To identify trends that may indicate naturally occurring or intentional disease outbreaks	In order to achieve early warning of health threats, early detection of health events, and overall situational awareness of disease activity	For early warning of threats and hazards, early detection of events,and rapid characterization of the event so that effective actions can be taken to mitigate adverse health, social, and economic effects	To achieve early detection and warning, contribute to overall situational awareness of the health aspects of an incident, and to enable better decision making at all levels

From the definitions in Table 4 it can be seen that the initial association of biosurveillance with syndromic surveillance placed biosurveillance as just one of many types of surveillance that could be used, depending on need (*e.g.* the NACHHO definition), for use in public health problems - in essence biosurveillance was one of public health surveillance's many tools. However, over time, the definition of biosurveillance has significantly broadened to take biosurveillance out of the context of being strictly a tool, to being a concept - similar in nature to the current understanding of public health surveillance (e.g. NBIC definition).

This two-way understanding of biosurveillance (as either a tool or as a concept) was also reflected in the survey responses of our SME panel. The majority of SMEs defined biosurveillance narrowly as surveillance for the *detection of disease outbreaks caused by infectious pathogens*. In 5 survey responses, however, the broader Homeland Security Presidential Directive 21(HSPD 21) definition of biosurveillance was given[36] (Table 4) (note, the National Strategy for Biosurveillance was not released at the time of the survey).

Viewing biosurveillance as an overarching concept rather than a tool was even more readily apparent when definitions were given for global and integrated biosurveillance. SME, global biosurveillance definitions typically expanded biosurveillance to include an international/worldwide perspective and cover human, animal and plant diseases across space, time, and geography while the definition of integrated biosurveillance included collaboration and cooperation among stakeholders.

An advantage in understanding biosurveillance as a concept is in removing biosurveillance from being one of the myriad types of surveillance that are frequently referred to as components of a surveillance system: active, passive, syndromic, clinical, traditional, non-traditional, sentinel, indicator-based, event-based, population-based, structured, unstructured, baseline, disease, epidemiologic, etc.

These surveillance modifiers [37] are descriptions that are more applicable to data sources than to surveillance as a whole[37]. As explained by Morse [38], these types of surveillance specifically associated with biosurveillance or public health surveillance are many times contrasted by their opposites and also are not mutually exclusive, which can be obfuscating rather than clarifying[37]. In our framework development, the above types of surveillance are considered in the data stream characterization component of our framework.

While it is unlikely that the current understanding of biosurveillance will remain unchanged, the concept definitions given in HSPD 21, and the National Strategy for Biosurveillance is the context that biosurveillance was understood in our development of the Biosurveillance Data Stream Framework.

Accordingly, in our framework the scope of biosurveillance is defined by the National Strategy [17].

#### Biosurveillance Goals

The interplay between objectives/goals and definition can cause confusion and as stated by Declich [39], “the objectives of surveillance are determined by the definition of surveillance being used”, because the objectives make up, in part, the biosurveillance definition, as shown in Table 3. Among the representative biosurveillance definitions given in Table 3, multiple goals are explicitly stated and range from identifying trends to rapid characterization of events and overall situational awareness.

Biosurveillance goals listed by the SME panel were also similar to those listed in Table 3. Frequently mentioned were early warning, early detection and situational awareness either broadly or with greater specificity (such as preventing disease spread for early warning). Additionally, SMEs considered the difference in goals from a military (force health protection) perspective versus a universal public health perspective.

Based on the multiple goals defined by the SME panel, the goals explicit in biosurveillance definitions, and through analysis of definitions in the literature, four broad biosurveillance goals that span the continuum of time were identified by us (Figure 1), and are based on terms that have been previously used by many in the public health and biosurveillance community.

**Early Warning of Health Threats:** Surveillance that enables identification of potential threats including emerging and re-emerging diseases that may be undefined or unexpected.

**Early Detection of Health Events:** Surveillance that enables identification of disease, outbreaks (either natural or intentional in origin), or events that have occurred, before they become significant.

**Situational Awareness:** Surveillance that monitors the location, magnitude, and spread of an outbreak or event once it has occurred

**Consequence Management**: Surveillance that assesses impacts and informs response to an outbreak or an event

**Baseline Awareness:** Information that can inform and facilitate the achievement of the above surveillance goals and can be related to population demographics and health, the natural, social, and built environment, and underlying disease patterns and characteristics.

Early event detection has been described as “gathering and analyzing data in advance of diagnostic case confirmation to give early warning of a possible outbreak and, should an outbreak exist, provide early detection”[27].

Consensus has not been reached regarding how the term situational awareness should be used in the context of public health surveillance or for biosurveillance. Situational awareness as broadly defined by Endsley [40], “perception of elements in the environment within a volume of time and space, the comprehension of their meaning, and the projection of their status in the near future” is the more common interpretation[41], [42]. However, situational awareness has also been put in the context of time - after an event has occurred- and the definition is narrowed to “real-time analysis and display of health data to monitor the location, magnitude, and spread of an outbreak.”[27] This more constrained definition allows for a distinction between situational awareness and background information. It is in this context that we are using situational awareness.

**Figure 2:**
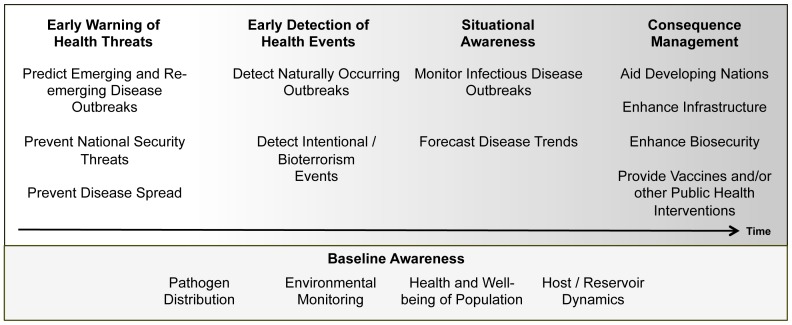
Biosurveillance goals identified by the SME panel and binned according to the biosurveillance goals defined in the Data Stream Framework.

Figure 2 illustrates these categorical goals and how goals given by the SMEs could be binned into these categories. While the goals follow a time-course, the absence of boundaries indicate that there is no absolute cut off on a time scale when any one surveillance goal would be deemed irrelevant. Likewise, Baseline Awareness is a significant requirement to achieve any of the surveillance goals identified in the figure.

It is important to note that in the context of our framework, biosurveillance is considered to be a concept that can be clarified by specifically defined goals that span time. Therefore, even if current definitions of biosurveillance, such as the one given in the National Strategy, are modified, the goals framework shown in Figure 1 can remain applicable across multiple changing biosurveillance definitions and is a key component of our Biosurveillance Data Stream Framework.

#### Data Stream Categorization

We developed a categorization scheme that would enable classification and categorization of individual data streams with the intent to be able to broadly categorize any individual data stream within the context of the scope and goals of biosurveillance. In this process a definition of a data stream for biosurveillance is defined, and examples of data streams are classified into the characterization framework.

Multiple data sources are used in a variety of biosurveillance systems that extend from a singular goal for local surveillance of a specific disease to wide-ranging surveillance systems requiring extensive data resources to meet diverse surveillance goals for an array of diseases. With the advent of new technologies, globalization, high performance computing, and ‘big data’ opportunities, there are seemingly unlimited potential data streams that could be useful in biosurveillance. Data streams have not been *universally* defined in either the literature or by specific systems. In order to develop an *explicit reference system* for data streams, multiple sources of information were gathered, categorized, and analyzed for best use in both describing the data streams and for database development, as described in the Methodology section.

Broad agreement from SME survey responses, defined a ‘data stream’ as a single source of information or data that could be used in a biosurveillance system. Collating the SME responses, a definition of an *ideal* data stream emerged:

“A single source of unique, timely (real-time), and spatially relevant information that is standardized and collected in a quantity and class that is needed for meaningful results, that targets a specific population, that is available at many scales (from molecular to ecosystem), is electronically available in both raw and reportable form, and has been rigorously validated.”

Unfortunately, using this definition, few, if any, data sources could be considered biosurveillance data streams. At issue is the difference between “data stream” and “data source”, with data stream being the more restrictive term by inherently implying a flow of information.

‘Data stream’ from a computational perspective has been defined as “a sequence of digitally encoded signals representing information when it is transmitted”[43]. Some biosurveillance data meet this criteria of digital encodement and have been described as data streams[44]–[47], such as the digital information pulled from electronic medical records or over-the-counter prescription drug sales. Other biosurveillance data do not: e.g., the astute observer calling in an alert. That alert, however, if picked up by the appropriate decision maker, becomes a part of the stream of information that informs the biosurveillance goal.

‘Data stream’ was also defined more broadly by several SMEs as “a continuous flow of information or data”. Under this broader definition a data source, therefore, might be or become a data stream depending on how the data is collected. This is readily apparent in the transformation of medical records from papers in files to digitally recorded information. The underlying information: the chief complaint, clinician's diagnosis, ordered prescriptions, and the associated meta data about the patient such as age, gender, and address has not changed. What has changed is the way the information is recorded, collected, accessed and subsequently analyzed. More and more, data sources are becoming available in some sort of digital form that could be collected and monitored. Even within a type of data source (such as medical records) some are available digitally and some are not. By understanding a data stream in this context - data that is available, or may become available for analysis in a biosurveillance system - the term data stream can be distinguished from data source, yet still retain the broad meaning of a flow of information.

For our purposes, once a data source has been identified as potentially having value in biosurveillance it can be considered a data stream, even if that stream might only come to exist in some future time.

**Figure 3:**
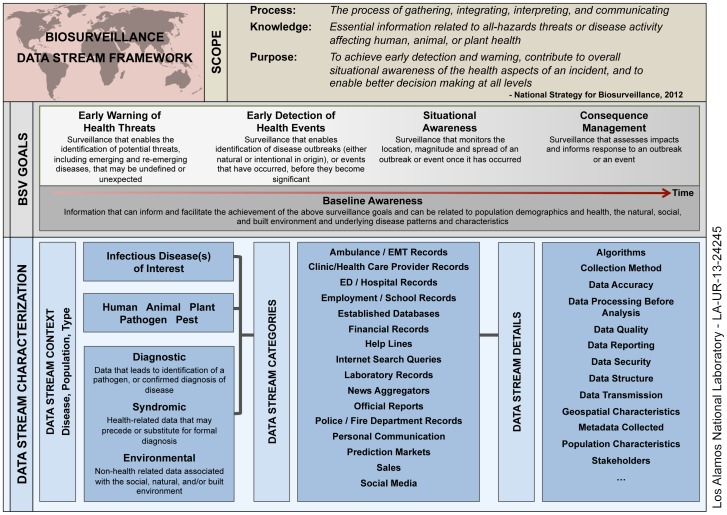
Biosurveillance Data Stream Framework.

The context in which the data stream categories are considered is shown in the complete Biosurveillance Data Stream Framework, Figure 3. The following terms and definitions were developed or adapted by us to facilitate a consistent approach to evaluation and to provide an invariant frame of reference:

**Biosurveillance Data Stream –** any data source that has been shown to have value, or might potentially have value to be collected and analyzed to inform a biosurveillance goal. A biosurveillance data stream is understood in the context of data population (human, animal, plant, pathogen, vector) and data type (environmental, syndromic, diagnostic) and is described by specific details such as data structure and collection method.

**Data Population** - The population that the data stream is associated with: human, animal (wild or domestic), plant, pathogen, vector

**Data Type** - Determined by the source of the data and is classified as environmental, syndromic or diagnostic.

- **Syndromic** -Health-related data that may precede or substitute for formal diagnosis[18] (non-specific health indicator data) [48]
- **Diagnostic** - Data that leads to the identification of a pathogen, or confirmed diagnosis of disease.
- **Environmental** - Pertaining to data streams that are not directly related to health parameters such as *social, built, and natural* environments. While other types of environments have been described such as political and economic, these are being grouped within the social environment. Within the built and natural environments there can be a great deal of interaction and overlap[49], but the broad distinction between them is the presence or absence of human development, and it is in that context that the definitions are put forth.
- *Social Environment* - the environment that includes the social, economic, and political conditions in which people live and work, and can include population demographics, population movements, and political and social engagement.
- *Natural Environment* - the non-manmade components of environments that can include climate and natural resources.[50]
- *Built Environment*- Distinct from the natural environment, the built environment is comprised of manmade components of people's surroundings, from small-scale settings (e.g., offices, houses, hospitals, shopping malls, and schools) to large-scale settings (e.g., neighborhoods, communities, and cities), as well as roads, sidewalks, green spaces, and connecting transit systems. [47], [48]

Population, Type, and Data Stream categories characterize *the kind of information that is being collected*, and how it could impact biosurveillance.

Also associated with each individual data stream (if the information is available) are data stream descriptors/details. These descriptors/details are specific to how the data is collected (mobile phones or surveys as shown in Table 5), how the data is structured, what geographic regions are covered, accessibility and update frequency. All of these descriptors inform the *quality and usefulness* of the specific data stream.

**Table 5:** Examples of Data Stream Collection Methods.

Cell/Mobile Phone
Crowdsourcing
Data Mining
Database Upload
Electronic Record Feed
Email
Internet/Web
Manual
Mobile Lab
Landline Phone
Photographic Images
Remote Sensing
Satellite
Sensors
SMS/Text Messaging
Surveys

### Demonstration of Framework

Sixteen data stream categories emerged from the iterative development process (Table 6). Table 6 provides definitions and examples of these categories and examples of how the data stream categories can be sub-categorized as needed such as clinic/health care provider records being subcategorized into physician provided or veterinary provided records. Of significance is the fact that we were able to bin any data stream into one of the 16 major categories, and are continually refining the sub-categories to enable comprehensive characterization. This table also illustrates how commonly referenced data streams could be binned into the 16 broad categories.

**Table 6:** Data Stream categories, sub-categories and examples.

DATA STREAM CATEGORY	Sub-Category (not inclusive)	Specific Examples	Common examples
Ambulance/EMT*Records			
Dispatch information which can include incident date, time, nature of call, and patient information			Ambulance/paramedic records
Clinic/Health Care Provider Records			
Record of patient (animal/human) information that can include symptoms, pharmacy orders, diagnoses, laboratory tests ordered and results received	Physician, Veterinary		Records from doctor's visits
ED*/Hospital Records			
Record of patient information that can include discharge/transfer orders, pharmacy orders, radiology results, laboratory results and any other data from ancillary services or provider notes	Military/Veteran Facilities		ED/Nurse triage records
Employment/School Records			
Information collected from schools or places of employment that can include, location, illness, absence, and activity reports regarding students or employees	Absenteeism, Illness, Activities		School nurse reports, Absentee data
Established Databases			
Any data repository from which information can be retrieved	Demographic data, Geographic data, Weather pattern/Meteorological data, Environmental data, Genetic sequencing	Google Earth, Google Maps, CIA Factbook, Toxnet, Census	Environmental data, Genomic data, Demographic data
Financial Records			
Records of financial activities of a person, business, or organization	Insurance/HMO billing, Bank Records		
Help Lines			
Telephone or cellular call-in services	Health/Medical, Poison Control, Professional, Emergency, Reporting/Complaint	911, Nurse Hotlines	Nurse call center, Poison control center, Consumer complaint logs
Internet Search Queries			
Search terms that a user enters into a web search engine	Global, Site Specific	Google, Yahoo	
Laboratory Records			
Information regarding specific tests ordered and/or the results of those tests	Laboratory Orders, Laboratory Results	PCR, Molecular Typing	Disease diagnostics, Pathogen diagnostics
News Aggregators			
Systematic collection of information from news sources that can include online and offline media	RSS feeds, Radio, Video, Newspapers, Press Releases, Media Monitoring	Google News	
Official Reports			
Any report that has been certified or validated from an authorized entity	Government, Intelligence, Industry, Non-profit, Academic	WHO, CDC/MMWR, Notifiable Disease, Peer Reviewed Literature	Environmental Reports, Epidemiological Reports
Police/Fire Department Records			
Dispatch and event information			
Personal Communication			
Any type of information that is directly relayed from one individual to another individual or group	Expert, Non-Expert		Public meetings, Case notes, Case studies
Prediction Markets			
Marketplaces for contracts in which the payoffs depend on the outcome of a future event	Health, Event	Iowa Electronic Health Markets	
Sales			
Monetary transactions for goods or services	Medical, Commercial	Drugs (OTC/Rx), Facial Tissue	Prescription sales, Grocery sales
Social Media			
Forms of electronic communication such as websites for social networking and blogging through which users create online communities to share information	Blogs, Internet Chatting, Social Networking Sites, Video-sharing	Facebook, MySpace, Twitter, YouTube	

EMT, emergency medical technician; ED, emergency department

#### Data Stream Examples

As an example in binning a data stream according to this framework, consider a data stream that monitors Google search queries for health-related key words. This data stream would be categorized as the following:

**Data Stream Context:** Population, Human; Type: Syndromic

**Data stream Category:** Internet search queries

In contrast if a data stream were monitoring Twitter for social unrest key words or phrases, the data stream would be categorized as:

**Data Stream Context:** Population, Human; Type, Environmental/Social

Data stream Category: Social Media

We subsequently applied our characterization schema to current and potential biosurveillance data streams that were identified by our SME panel as 'non-traditional'. How those data streams would be placed into our sixteen categories within the context of data population and data type is shown in Table 7. A total of 34 specific but disparate 'non-traditional' data streams were characterized using our framework. Evident in Table 7 is that many of the non-traditional data sources that were enumerated by our SME panel are already being used, indicating that 'non-traditional' is a variable concept and data streams that may be considered 'non-traditional' will depend on the individual perspective of the responder. An advantage of binning the data streams in our framework is to avoid the vague connotation of the term 'non-traditional' and replace this with specifically defined terminology.

**Table 7:** SME Non-traditional data streams binned according to the Biosurveillance Data Stream Framework.

SME	Population	Type	Category	Detail
Syndromic/observational, chief complaint	Human	Syndromic	ED/Hospital Records, Clinic/Health Care Provider	Chief Complaint
Nurse call center	Human	Syndromic	Help Lines	Nurse call center
EMS 911 calls	Human	Syndromic	Help Lines	911
School nurse illness reports	Human	Syndromic	School Records	Illness
Drug trends, OTC sales	Human	Syndromic	Sales	Drugs
Ambulance dispatch records	Human	Syndromic	Ambulance Records	
ED/Nurse triage notes	Human	Syndromic	ED/Hospital Records	
EMR - narrative text	Human	Syndromic	ED/Hospital Records	Collection Method: Data Mining
Doctors visits	Human	Syndromic	Clinic/Health Care Provider	Individual Case Reports
Practitioner information regarding consultations or investigations	Human	Syndromic	Clinic/Health Care Provider	Individual Case Reports, Aggregate Case Reports
Poison Control Center - narrative text	Human	Syndromic	Help Lines	Poison Control, Collection Method:Data Mining
Chemical and radiological exposure data	Human	Diagnostic	Official Reports, Established Databases	
Host susceptibility to health threats	Human, Animal, Plant	Environmental/Social	Official Reports, Established Databases	
Absentee data	Human	Syndromic	School/Employment Records	Absenteeism
School activities	Human	Environmental/Social	School/Employment Records	Activities
Grocery purchase trends, purchasing trends	Human	Syndromic, Or Environmental/Social	Sales	Groceries
Consumer complaint logs	Human	Syndromic, Or Environmental/Social	Sales	Complaints
Bank saving withdraws	Human	Environmental/Social	Financial Records	
Public meetings	Human	Syndromic, Or Environmental/Social	News Aggregators	
Food Trade	Human	Syndromic, Or Environmental/Social	Official Reports, Financial Records	Food Trade
Grouping and evaluating laboratory testing data to include trend analysis of types of requested diagnostic tests as an indication of increased disease incidence	Human, Animal, Plant	Diagnostic	Laboratory Records	Data Processing
Genetic shift (bacterial, viral), terrestrial microbial genomics, genotype, phenotype, proteome, omics	Pathogen	Environmental/Social	Established databases, Official Reports	
Pathogen monitoring	Pathogen	Environmental/Social	Established Databases, Laboratory Records	Pathogen monitoring
Epidemiology investigator's case notes	Human, Animal	Syndromic	Personal Communication	Collection method
Population density shifts in humans and animals	Human, Animal	Environmental/Social	Established databases, Official Reports (academic)	
Human and animal behavior/events	Human, Animal	Environmental/Social	Official Reports (academic)	Human and animal behavior/events
Climate change, meteorology	All	Environmental/Natural	Established Databases, Official Reports	
Environmental Factors, data	All	Environmental/Natural	Established Databases, Official Reports	Environmental Factors, data
Water quality reports	All	Environmental/Built, Environmental/Natural	Official Reports	Water quality reports
Social unrest/disruption	Human	Environmental/Social	News Aggregators, Official Reports	
Mainstream News, open source reporting media, popular news outlets, magazines, radio, newsfeeds	Human	Environmental/Social, Environmental/Built, Syndromic	News Aggregators	
Social Media Traffic	Human	Environmental/Social, Syndromic	Social Media	
Crowd sourcing and social networks	Human	Environmental/Social, Syndromic	Social Media	
Intelligence reports	Human	Environmental/All, Syndromic, Diagnostic	Official Reports	Intelligence reports

A comparative analysis between disparate data streams is also enabled through our framework by analysis of the data stream details associated with each data stream. For instance, as described by Scharlemann et al., temperature measurements can be derived from ground-based meteorological records or through remotely sensed data from Earth-orbiting satellites[51], details associated with how the data is collected. The context and kind of information (temperature measurements) for both of these data streams are the same; Context: environmental/natural Data Stream Category: established database - but the details vary based on how the information is collected and transmitted, how often the measurements are made, what geographical region the measurements cover, and the granularity of the measurements. Accordingly, for a data stream coming from online surveys, the context would depend on the information being collected (disease, data population, data type). The category would be personal communication, and then the details would include how the information was collected (online). Such comparisons are difficult without systematic and structured descriptions as shown in the framework.

Finally, the choice and effectiveness of types of data streams should be informed by the specific goal(s) of the biosurveillance system, and by the diseases being monitored. These categories complete the framework for understanding data stream relevance that is shown in Figure 3.

#### Biosurveillance Resource Directory

An advantage of deconstructing data streams is in the ability to discuss data streams in broad terms yet still retain the detailed information that may be very important regarding actual utility of specific data sources. We used this framework in building the relational database described in the methodology section to categorize information on operational infectious disease biosurveillance systems. One example of the functionality and utility of the framework for understanding biosurveillance systems is the ability to categorize the systems based on multiple specific framework parameters. Individual data streams used by biosurveillance systems were categorized both broadly according to the framework, and in as much detail as needed or required in the database.

An example from the BRD is the system NCDetect, the North Carolina Disease Event Tracking and Epidemiologic Collection Tool[52]. A broad and detailed range of information was collected about NC Detect in the BRD. Data streams used by this system include emergency department data, poison center data, ambulance/EMS and urgent care center data. Reports from NC Detect include syndrome counts for selected syndromes and locations[52]. Information related to data streams was organized and categorized according to our framework:

**BSV Goal:** Early Detection

**Data Stream Context:**

Data Population: Human

Data Type: Syndromic

**Data Stream Categories:**

ED/Hospital Records

Help Lines (subcategory, poison control)

Ambulance/EMT Records

Clinic/Health Care Provider Records

In this example the BSV goal and data stream context was the same for all data streams, in the BRD, the data stream context and specific details about each data stream are cataloged independently.

**Figure 4:**
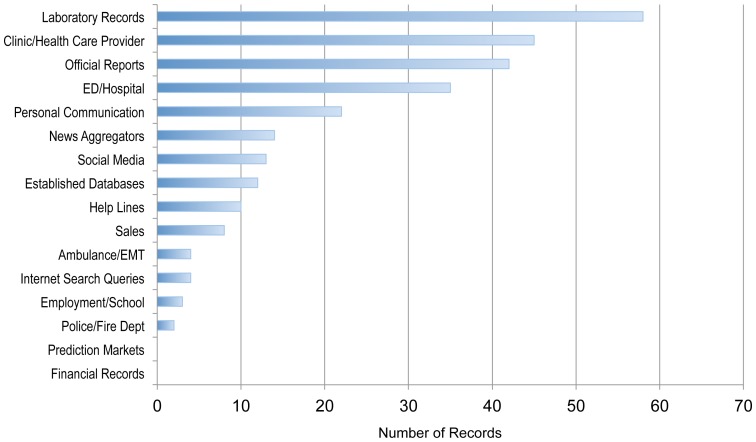
Data streams used in active operational biosurveillance systems as collected and categorized in the Biosurveillance Resource Directory (BRD). Data streams from 115 systems were tallied (some systems using more than one category of data stream).

From the resources cataloged in the BRD a total of 115 systems were found to meet our definition of a system. From these systems 272 data streams were recorded and tallied and are shown in Figure 4. The most commonly used data streams were laboratory records and clinic/health care provider records. While prediction markets and financial records have been described as potentially useful data streams for biosurveillance, they were not yet part of a currently active biosurveillance system that we have cataloged.

### Application of Framework

As described, we are using the BSV Data Stream Framework in the BRD, cataloging biosurveillance systems, data sources and tools. As the BRD is updated to include the most relevant biosurveillance resources, the data stream framework will be tested and refined to keep pace with the changing and evolving biosurveillance practices and associated data sources. Additionally, we will also be able to monitor the real-time use of data streams for different diseases, locations and purposes.

As an extension of the BRD, the current state-of-the-art in operational epidemiological modeling is also being cataloged by our team[53]. By binning specific data streams used as data inputs by the models into the Data Stream Framework, we can cross-compare models based on data sources and model objective in the context of biosurveillance goals.

Additionally, our team is using the Data Stream Framework in development of a multi-criteria decision analysis tool for evaluating specific novel data streams that become available for local, state, national and international disease surveillance as well as data streams currently in use as a means to provide a “real world” assessment of the data stream categories. Through multi-criteria decision analysis the advantages and disadvantages of determining which data streams to include in a biosurveillance system are considered through a systematic method for evaluating alternatives (data streams) based on a series of attributes (metrics) in the context of the associated biosurveillance goal. Our team has used this tool in analysis of categories of data streams[54], and work is ongoing for specific data stream evaluation.

Finally, the BSV Data Stream Framework was adopted for ground-truthing specific data streams used in selected infectious disease outbreaks. Outbreak timelines based on historical epidemiological data were developed for multiple disease-specific case studies. A window of time (the surveillance window) within which various data streams could be used to alert, or provide information about the unfolding outbreak, was identified, and outbreak-specific data streams that were available at that time were recorded [55]. By using the BSV data stream framework in combination with the surveillance window methodology[29], data streams of importance for early warning, early detection, and for containment of an outbreak were identified. Our team continues to expand our analysis of historical outbreaks and ground-truthing data streams for priority infectious diseases.

## Conclusions

The Biosurveillance Data Stream Framework and definitions that we are presenting provide a characterization methodology that facilitates understanding of data streams and allows for comparative analysis. The resulting framework puts data streams into context and provides for a systematic categorization such that ongoing discussion and debate regarding biosurveillance for particular diseases, events, and populations can lead to productive and practicable evaluation. We believe this framework can be useful to public health practitioners, biosurveillance analysts, surveillance system developers among others.

The foundation underlying this framework is both *novel*, because it has not been done before as comprehensively, iteratively, or categorically, and *timely*, because the many individual data streams that might by considered for biosurveillance can now be systematically categorized to enable comparison and analysis. We propose that this framework and associated definitions can serve as a foundation to build a standard lexicon for the growing field of biosurveillance.

## Supporting Information

Table S1 **Biosurveillance data stream examples reported in the literature and binned according to the Data Stream Framework.**
(PDF)

## References

[1] Khan K, Freifeld CC, Wang J, Mekaru SR, Kossowsky D, et al (2010) Preparing for infectious disease threats at mass gatherings: the case of the Vancouver 2010 Olympic Winter Games. Can Med Assoc J 182: 579–583 10.1503/cmaj.100093 20181726 PMC2845686

[2] Hardiman MW (2012) World Health Organization Perspective on Implementation of International Health Regulations. Emerg Infect Dis 18 . doi:10.3201/eid1807.120395. PMC3376823 22709544

[3] Taboy CH, Chapman W, Albetkova A, Kennedy S, Rayfield MA (2010) Integrated Disease Investigations and Surveillance planning: a systems approach to strengthening national surveillance and detection of events of public health importance in support of the International Health Regulations. BMC Public Health 10 Suppl 1S6 10.1186/14712458-10-s1-s6 21143828 PMC3005578

[4] Morse SS, Mazet JA, Woolhouse M, Parrish CR, Carroll D, et al (2012) Prediction and prevention of the next pandemic zoonosis. The Lancet 380: 1956–1965 10.1016/S0140-6736(12)61684-5 PMC3712877 23200504

[5] Bravata DM, McDonald KM, Smith WM, Rydzak C, Szeto H, et al (2004) Systematic review: Surveillance systems for early detection of bioterrorism-related diseases. Ann Intern Med 140: 910–922. 15172906 10.7326/0003-4819-140-11-200406010-00013

[6] Kloeze H, Mukhi S, Kitching P, Lees VW, Alexandersen S (2010) Effective Animal Health Disease Surveillance Using a Network-Enabled Approach. Transbound Emerg Dis 57: 414–419 10.1111/j.1865-1682.2010.01166.x 20846188

[7] Scott AE, Forsythe KW, Johnson CL (2012) National animal health surveillance: Return on investment. Prev Vet Med 105: 265–270 10.1016/j.prevetmed.2012.01.007 22305661

[8] Bradley CA, Rolka H, Walker D, Loonsk J (2005) BioSense: implementation of a National Early Event Detection and Situational Awareness System. Morb Mortal Wkly Rep 54 Suppl11–19. 16177687

[9] Drewe JA, Hoinville LJ, Cook AJ, Floyd T, Stark KD (2012) Evaluation of animal and public health surveillance systems: a systematic review. Epidemiol Infect 140: 575–590 10.1017/s0950268811002160 22074638

[10] Hartley DM, Nelson NP, Walters RA, Arthur R, Yangarber R, et al.. (2010) Landscape of International Biosurveillance. Emerg Heal Threats J. doi:10.3134/ehtj.10.003. PMC3167659 22460393

[11] Holtry R, Hung L, Lewis S (2010) Utility of Essence Surveillance in Monitoring the H1N1 Outbreak. Online J Public Heal Informatics 2. Available: http://www.ncbi.nlm.nih.gov/pmc/articles/PMC3615770/. Accessed 1 Nov 2013. 10.5210/ojphi.v2i3.3028 PMC3615770 23569593

[12] Hulth A, Rydevik G (2011) GET WELL: an automated surveillance system for gaining new epidemiological knowledge. BMC Public Health 11 . doi:10.1186/1471-2458-11-252. PMC3098167 21510860

[13] Lucero CA, Oda G, Cox K, Maldonado F, Lombardo J, et al. (2011) Enhanced health event detection and influenza surveillance using a joint Veterans Affairs and Department of Defense biosurveillance application. BMC Med Inform Decis Mak 11 . doi:10.1186/1472-6947-11-56. PMC3188469 21929813

[14] Lyon A, Nunn M, Grossel G, Burgman M (2011) Comparison of Web-Based Biosecurity Intelligence Systems: BioCaster, EpiSPIDER and HealthMap. Transbound Emerg Dis: 223–232. doi:10.1111/j.1865-1682.2011.01258.x. 22182229

[15] Rolka H, O'Connor J (2011) Real-Time Public Health Biosurveillance; In: Castillo-Chavez CC, editor. Infectious Disease Informatics and Biosurveillance. Integrated Series in Information Systems. Springer US, Vol. 27. pp. 3–22. Available: 10.1007/978-1-4419-6892-0_1.

[16] Russell KL, Rubenstein J, Burke RL, Vest KG, Johns MC, et al (2011) The Global Emerging Infection Surveillance and Response System (GEIS), a U.S. government tool for improved global biosurveillance: a review of 2009. BMC Public Health 11 Suppl 2S2 10.1186/1471-2458-11-s2-s2 PMC3092412 21388562

[17] White House (2012) National Strategy for Biosurveillance. Available: http://www.whitehouse.gov/sites/default/files/National_Strategy_for_Biosurveillance_July_2012.pdf. Accessed 1 Nov 2013.

[18] ICAHS (2011) Animal Health Surveillance Terminology, Draft output from pre-ICAHS workshop. Available: http://animalhealthsurveillance.org/index.php?n=Main.TerminologyFinal. Accessed 1 Nov 2013.

[19] Chute CG (2008) Biosurveillance, classification, and semantic health technologies. J Am Med Inform Assoc 15: 172–173 10.1197/jamia.M2693 18396506 PMC2274785

[20] Kman NE, Bachmann DJ (2012) Biosurveillance: a review and update. Adv Prev Med 2012: 301408 10.1155/2012/301408 22242207 PMC3254002

[21] Hitchcock P, Chamberlain A, Van Wagoner M, Inglesby TV, O'Toole T (2007) Challenges to global surveillance and response to infectious disease outbreaks of international importance. Biosecurity Bioterrorism-Biodefense Strat Pr Sci 5: 206–227 10.1089/bsp.2007.0041 17903090

[22] Parry B (2012) Domesticating biosurveillance: “Containment” and the politics of bioinformation. Health Place 18: 718–725 10.1016/j.healthplace.2011.10.010 22682087

[23] Corley CD, Lancaster MJ, Brigantic RT, Chung JS, Walters RA, et al.. (2012) Assessing the Continuum of Event-Based Biosurveillance Through an Operational Lens. Biosecur Bioterror. doi:10.1089/bsp.2011.0096. 22320664

[24] Malecki KC, Resnick B, Burke TA (2008) Effective Environmental Public Health Surveillance Programs: A Framework for Identifying and Evaluating Data Resources and Indicators. J Public Health Manag Pract 14: 543–551 10.1097/01.PHH.0000338366.74327.c9 18849774

[25] Sosin DM (2003) Draft framework for evaluating syndromic surveillance systems. J Urban Heal-Bull New York Acad Med 80: I8–I13. 10.1007/PL00022309 PMC3456539 12791773

[26] German RR, Lee L, Horan J, Milstein R, Pertowski C, et al (2001) Updated guidelines for evaluating public health surveillance systems: recommendations from the Guidelines Working Group. Morb Mortal Wkly Rep 50: 1–35. 18634202

[27] Fricker RD (2011) Some methodological issues in biosurveillance. Stat Med 30: 403–415 10.1002/sim.3880 21312208

[28] Buckeridge DL (2007) Outbreak detection through automated surveillance: a review of the determinants of detection. J Biomed Inform 40: 370–379 10.1016/j.jbi.2006.09.003 17095301

[29] Deshpande A, Brown M, Castro L, Daniel W, Generous E, et al. (2013) A Systematic Evaluation of Traditional and Non-Traditional Data Streams for Integrated Global Biosurveillance – Final Report. Los Alamos National Laboratory. Available: http://www.osti.gov/bridge/purl.cover.jsp?purl=/1077029/. Accessed 1 Nov 2013.

[30] Wagner ML, Carter CC, Lewis SL, Feighner BH (2011) Human Public Health Surveillance Systems Catalog. The Johns Hopkins University; Applied Physics Laboratory

[31] National Public Health Surveillance and Biosurveillance Registry for Human Health (2011) Centers for Disease Control and Prevention.

[32] Buehler JW, Berkelman RL, Hartley DM, Peters CJ (2003) Syndromic surveillance and bioterrorism-related epidemics. Emerg Infect Dis 9: 1197–1204 10.3201/eid0910.030231 14609452 PMC3033092

[33] Henning K (2004) Overview of Syndromic Surveillance: What is Syndromic Surveillance? Morb Mortal Wkly Rep 53 (Suppl): 5–11.

[34] Weber TE (2001) Computers Could Help Health Officials Detect Bioterrorist Attacks. Wall Str J East Ed: B.1.

[35] Thacker SB, Berkelman RL (1992) History of Public Health Surveillance. In: Halperin W, Baker EL, Monson RR, editors.Public Health Surveillance. John Wiley & Sons, Inc.

[36] U.S. Department of Homeland Security (2007) HSPD 21: Public Health and Medical Preparedness. Available: http://www.fas.org/irp/offdocs/nspd/hspd-21.htm. Accessed 8 April 2013.

[37] Thacker SB, Qualters JR, Lee LM (2012) Public Health Surveillance in the United States: Evolution and Challenges*. Morb Mortal Wkly Rep 61: 3–9. 22832990

[38] Morse SS (2012) Public health surveillance and infectious disease detection. Biosecur Bioterror 10: 6–16 10.1089/bsp.2011.0088 22455675

[39] Declich S, Carter AO (1994) Public health surveillance: historical origins, methods and evaluation. Bull World Health Organ 72: 285–304. 8205649 PMC2486528

[40] Endsley MR (1995) Toward a Theory of Situation Awareness in Dynamic Systems. Hum Factors J Hum Factors Ergon Soc 37: 32–64 10.1518/001872095779049543

[41] Concept Plan for Implementation of the National Biosurveillance Strategy for Human Health (2010) U.S. Department of Health and Human Services. Available: www.cdc.gov/osels/pdf/Concept_Plan_V15finalforprintKMD.PDF. Accessed 1 Nov 2013.

[42] National Biosurveillance Integration Center Strategic Plan (2012) Department of Homeland Security. Available: https://www.dhs.gov/publication/nbic-strategic-plan. Accessed 1 Nov 2013.

[43] Data Stream Definition (n.d.). Dictionary.com. Available: http://dictionary.reference.com/browse/data_stream. Accessed 5 November 2013.

[44] Walters RA, Harlan PA, Nelson NP, Hartley DM, Voeller JG (2010) Data Sources for Biosurveillance. Wiley Handbook of Science and Technology for Homeland Security.Inc. John Wiley & Sons, Vol. 4 .pp. 2431–2447. Available: 10.1002/9780470087923.hhs151.

[45] Dixon BE, McGowan JJ, Grannis SJ (2011) Electronic laboratory data quality and the value of a health information exchange to support public health reporting processes. AMIA Annual Symp Proc Vol. 2011 . pp. 322–330. PMC3243173 22195084

[46] Mostashari F, Fine A, Das D, Adams J, Layton M (2003) Use of ambulance dispatch data as an early warning system for communitywide influenzalike illness, New York City. J Urban Heal 80: i43–9. 10.1007/PL00022314 PMC3456514 12791778

[47] Brownstein JS, Freifeld CC, Madoff LC (2009) Digital disease detection—harnessing the Web for public health surveillance. N Engl J Med 360: : 2153–2155, 2157. doi:10.1056/NEJMp0900702. PMC2917042 19423867

[48] Heffernan R, Mostashari F, Das D, Karpati A, Kulldorff M, et al (2004) Syndromic Surveillance in Public Health Practice, New York City. Emerg Infect Dis 10: 858–864 10.3201/eid1005.030646 15200820

[49] Our Built and Natural Environments (n.d.). Available: http://www.epa.gov/smartgrowth/built.htm. Accessed 13 June 2013.

[50] Johnson DL, Ambrose SH, Bassett TJ, Bowen ML, Crummey DE, et al (1997) Meanings of environmental terms. J Environ Qual 26: 581–589.

[51] Scharlemann JPW, Benz D, Hay SI, Purse BV, Tatem AJ, et al (2008) Global Data for Ecology and Epidemiology: A Novel Algorithm for Temporal Fourier Processing MODIS Data. PLoS ONE 3: e1408 10.1371/journal.pone.0001408 18183289 PMC2171368

[52] NC DETECT: North Carolina Disease Event Tracking and Epidemiologic Collection Tool (n.d.). Available: http://www.ncdetect.org/index.html. Accessed 5 September 2013.

[53] Deshpande A, Margevicius K, Castro L, Taylor-McCabe K, Generous EN, et al.. (2014) Tools and Apps to Enhance Situational Awareness for Global Disease Surveillance. Online Journal of Public Health Informatics. Vol. in press.

[54] Generous EN (2013) Evaluating Biosurveillance System Components using Multi-Criteria Decision Analysis. Online J Public Heal Informatics 5 . doi:10.5210/ojphi.v5i1.4508.

[55] McCabe K (2013) The Surveillance Window - Contextualizing Data Streams. Online J Public Heal Informatics 5 . doi:10.5210/ojphi.v5i1.4404.

[56] Thacker SB, Berkelman RL (1988) Public health surveillance in the United States. Epidemiol Rev 10: 164–190. 3066626 10.1093/oxfordjournals.epirev.a036021

[57] Hall HI, Correa A, Yoon PW, Braden CR (2012) Lexicon, definitions, and conceptual framework for public health surveillance. Morb Mortal Wkly Rep 61: 10–14. 22832991

[58] Lee LM, Thacker SB (2011) Public Health Surveillance and Knowing About Health in the Context of Growing Sources of Health Data. Am J Prev Med 41: 636–640 10.1016/j.amepre.2011.08.015 22099242

[59] Seiders B, Rybka A (2002) Technologies for distributed defense. . . .Proceedings of SPIE. Vol. 4745 . p.13. Available: http://proceedings.spiedigitallibrary.org/data/Conferences/SPIEP/31583/13_1.pdf. Accessed 14 May 2013.

[60] Wagner MM, Moore AW, Aryel RM (2006) Handbook of biosurveillance. Elsevier Press.

[61] NACCHO Positions | NACCHO (2011) Available: http://www.naccho.org/advocacy/positions/.Accessed 1 March 2013.

[62] Kaydos-Daniels SC, Rojas Smith L, Farris TR (2013) Biosurveillance in Outbreak Investigations. Biosecurity Bioterrorism Biodefense Strat Pr Sci: 20–28. doi:10.1089/bsp.2011.0109. 23448272

