# Supplementary material for: Advancing a Framework to Enable Characterization and Evaluation of Data Streams Useful for Biosurveillance
Source: PLoS One. 2014 Jan 2;9(1):e83730. doi: 10.1371/journal.pone.0083730 (PMC3879288; doi:10.1371/journal.pone.0083730)
Supplement: Table S1 — Biosurveillance data stream examples reported in the literature and binned according to the Data Stream Framework. (PDF) [file pone.0083730.s001.pdf]

Table S1. Biosurveillance data stream examples reported in the literature and binned according to the Data Stream Framework.

Population	Type	Category	Descriptors	Reference
Human	Diagnostic	Laboratory Records	Monitored malaria case loads	[1]Mueller 2009
Animal, Plant, Human	Diagnostic	Laboratory Records Official Reports	Optimal sentinel data	[2]Polgreen 2009
Human	Environmental Syndromic	Social Media	Insights for Search - Google	[3]Scheitle 2011
Human	Syndromic	Ambulance / EMT Records	Dispatches	[4]Plagianos2011, [5]Buehler 2008, [6]Hope 2010
Human	Diagnostic/Syndromic	Clinic / Health Care Provider Records	Biosense-CDC	[7]Tokars 2010, [8]Bradley 2005
Human	Diagnostic/Syndromic	Clinic / Health Care Provider Records	Clinician based reporting	[9]Koski 2011
Human	Diagnostic/Syndromic	Clinic / Health Care Provider Records	Timeliness of records	[10]Huaman 2009
Human	Diagnostic/Syndromic	Clinic / Health Care Provider Records	Health Care professionals	[11]Ashford 2003
Animal	Diagnostic/Syndromic	Clinic / Health Care Provider Records	Veterinary	[12]Thomas-Bachil 2012, [13]Bartlett 2010
Human	Syndromic	ED/ Hospital Records	ED visits, chief complaints	[6]Hope 2010
Human	Syndromic	ED/ Hospital Records	Hospitals, inpatient: chief complaint	[7]Tokars 2010
Human	Syndromic Diagnostic	ED/ Hospital Records	Hospital, ED ICD-9 codes	[14]Shmueli 2010
Human	Diagnostic	ED/ Hospital Records	Emergencey department diagnosis	[4]Plagianos2011
Human	Diagnostic	ED/ Hospital Records	DoD Outpatient: final Diagnosis	[7]Tokars 2010
Human	Diagnostic	ED/ Hospital Records	VA Outpatient: final Diagnosis	[7]Tokars 2010
Human	Diagnostic	ED/ Hospital Records	Hospitals, inpatient: final diagnosis	[7]Tokars 2010
Human	Diagnostic/Syndromic	ED/ Hospital Records	Clinic or Emergency Department (ED) visits	[5]Buehler 2008
Human	Syndromic	ED/ Hospital Records	Syndromic hospital surveillance	[7]Tokars 2010, [6]Hope 2010, [4]Plagianos 2011 [15]Malik 2011
Animal, Plant, Human	Diagnostic/Syndromic	ED/ Hospital Records Clinic/Health Care Provider Records	Electronic health records Veterinary	[16]Rabadan 2009
Human	Syndromic	Employment/School Records	School absenteeism	[5]Buehler 2008 [17]Balabanova 2011
Human	Syndromic	Employment/School Records	Work absenteeism<br><br>School nurse	[5]Buehler 2008 [17]Balablanova 2011, [8]Bradley 2005
Animal	Environmental/Social	Established database	Wildlife Tracking	[18]Freifeld 2010
Animal	Environmental/Social	Established Database	Remotes Sensing	[19]Clements 2009

Pathogen			Geo location and remote sensing of viral zoonosis	
Human	Environmental/Social -Built-Natural	Established Database	CIA Factbook	[20]Keller 2009
All	Environmental/Natural	Established Database	Solar radiation, dew point, temperature	[21]Charland 2009, [22]Degroote 2008
All	Environmental/Natural	Established Database	Climate monitoring (malaria)	[23]Cox 2007
All	Environmental/Natural	Established Database	Meteorological factors	[24]Kristan 2008
All	Environmental/Natural	Established Database	Seasonal climate forecasts	[1]Mueller 2009
All	Environmental/Natural	Established Database	Observed meteorlogical data	[1]Mueller 2009
All	Environmental/Social	Established Database	Demographics	[25]Chui 2011
All	Environmental/Natural	Established Database	Geospatial analysis	[26]Baker 2011
All	Environmental/Natural	Established Database	Geographical position	[[21]Charland 2009
All	Environmental/Natural	Established Database	Google Maps/Google Earth	[27]Atkinson 2008
Human	Syndromic	Help Lines	Health/Medical hotline calls	[8]Bradley 2005 [5]Buehler 2008
Human	Syndromic	Help Lines	Poison Control Center calls	[5]Buehler 2008
Human	Syndromic	Help Lines	Emergency medical services (EMS)/911 calls	[5]Buehler 2008
Human	Environmental/Built	Help Lines	Water utility complaint lines, call center	[28]Berger 2006
Human	Syndromic	Internet Search Queries	Google flu trends -internet searches	[29]Ortiz 2011
Human	Syndromic	Internet Search Queries	Search engine queries (ILI)	[30]Ginsberg 2009
Human	Environmental Syndromic	Internet Search Queries	Web-based search queries	[31]Hulth 2011, [32]Brownstein 2008, [33]Brownstein 2009, [34]Chan 2011, [35]Hulth 2010, [36]Polgreen 2008, [37]Madoff 2011
Human	Syndromic	Internet Search Queries	Anonymous web query data - from country medical sites	[31]Hulth 2011
Animal	Diagnostic	Laboratory Reports	Zoo - necropsies	[38]McNamara2007
Animal	Syndromic<br>Diagnostic	Laboratory Reports	Veterinary syndromic surveillance<br>-clinical data from practitioners<br>-diagnostic laboratory data	[39]Dorea 2011
Animal	Diagnostic	Laboratory Reports	Veterinary and Zoo reporting - Data elements required for lab submission for disease reporting that allow for voluntary collaboration and data sharing by avoiding issues associated with privacy legislation	[40]Kloeze 2011

Human, Animal	Diagnostic	Laboratory Reports	Food sample testing	[41]Dubrawski 2009
Human, Animal, Pathogen	Diagnostic	Laboratory Reports	Biotracing biological contamination in feed/food chain	[42]Hoorfar 2011, [43]Knutsson 2011
Pathogen	Diagnostic	Laboratory Reports	Molecular diagnostic reporting Molecular techniques for pathogen identification Genotyping	[44]Olano 2011 [16]Rabadan 2009 [45]Sintchenko 2009, [26]Baker 2011
Human	Diagnostic	Laboratory Reports	Breath Analyzer for real-time VOC (Volatile organic compounds) Mobile	[46]Churchill 2009
Human	Diagnostic	Laboratory Reports	GPS enabled inhaler + iphone Mobile	[18]Freifeld 2010
Human	Diagnostic	Laboratory Reports	Mobile phone - geocoded photographs of blood samples for rapid detection of malaria strains Mobile l	[47]Fuller 2010
Animal, Plant, Human	Diagnostic	Laboratory Reports	Sentinel health facilities -electronic transfer of data by mobile phones -manual transfer of data	[1]Mueller 2009
Human	Syndromic	News Aggregators	Online health news alerts	[48]Collier 2010
Human	Syndromic	News Aggregators	Internet news feeds unstructured and structured data sets	[49]Grady 2008
Human	Environmental Syndromic	News Aggregators	Online news monitoring	[27]Atkinson 2008, [33]Brownstein 2009
Human	Environmental Syndromic	News Aggregators	Google news	[50]Keller 2009, [32]Brownstein 2008
Human	Environmental Syndromic	News Aggregators	RSS feeds	[50]Keller 2009
Human	Environmental Syndromic	News Aggregators	Web news sites plus natural language processing to transform free text into structured info stored in a relational data base with GIS tags	EpiSpider [50]Keller 2009
Human	Environmental Syndromic	News Aggregators	Online news articles	[51]Torii 2011
All	Environmental/Social -Natural	News Aggregators	Violent and natural disaster event extraction from online news	[52]Piskorski 2011
Human	Environmental/Social -Natural	News Aggregators	Real time news event extraction with limited linguistic sophistication for violent and disaster events	[53]Tanev 2008
Human	Environmental/Social	News Aggregators Social Media	Internet indicators and warnings of social disruption	[54]Wilson 2009
Human	Environmental Syndromic	News Aggregators Social Media	Unverified news information	[55]Mykhalovskiy 2007

			Mainstream news	
Human	Environmental<br>Syndromic	News Aggregators<br>Social Media	Text mining (ontology based)<br>Mainstream news	[56]Drague 2010
Animal	Environmental/Social	Official Reports	Zoo- report pest control measures on grounds<br>Zoo- survey of local wildlife on grounds	[38]McNamara2007
Animal	Syndromic	Official Reports	Monitoring of sentinel bird flocks - detect and report dead bird clusters in real time	[19]Clements 2009
Animal	Syndromic<br>Diagnostic	Official Reports	Zoos - sentinel animal data	[57]Pultorak 2011, [58]Adler 2011
Human	Environmental/Social	Official Reports	UN Human Development Report	[50]Keller 2009
Human	Environmental/Built	Official Reports	Drinking water turbidity levels<br>Environmental case report	[28]Berger 2006
Human	Environmental/Built	Official Reports	Public utilities data	[59]Baer 2011
Pathogen	Environmental/Natural	Official Reports	Geographic distribution of viruses<br>Remote sensing	[60]Trifonov 2009
All	Environmental/Natural -Built	Official Reports	Air quality<br>Established monitoring system	[61]Babin 2008
All	Environmental/Natural -Built	Official Reports	Water quality<br>Established monitoring system	[28]Berger 2006, Burkom 2011
Pathogen	Environmental/Social	Official Reports<br>Academic	Disease vector populations	[58]Adler 2010
Pathogen<br>Animal	Environmental/Social	Official Reports<br>Academic	Vector densities	[24]Kristan 2008
Pathogen	Environmental/Natural	Official Reports<br>Academic	Pyhlogeography(spatial analysis of genetic variation of pathogens)	[19]Clements 2009
Human	All	Official Reports<br>Laboratory Records	Multinational surveillance reports (surveillance systems)<br>Established surveillance system	[50]Keller 2009
Animal	Environmental/Social	Official Reports<br>Academic	Geographic patterns of livestock/animal population distributions<br>Animal/herd movement	[62]Ortiz-Pelaez 2010 [63]Vernon 2009
Human	Diagnostic	Official Reports	Validated official alerts (WHO, CDC)	[20][50]Keller 2009, [64]Briand 2011
	Diagnostic	Official Reports	Mortality reports	
Animal	Syndromic<br>Diagnostic	Personal Communication<br>Official Reports	Livestock monitoring<br>- web-based data set to share results with privacy controls	[40]Kloeze 2011
Human	Syndromic<br>Envrionmental/Social	Prediction Markets	Prediction markets	[65]Polgreen
Human	Syndromic	Prediction Markets	Prediction markets (experts, health care	[66]Pfeiffer 2010, [65]Polgreen 2007

			providers)	
Human	Syndromic	Sales	Over-the-Counter (OTC) medicine purchases	[28]Berger 2006 [5]Buehler 2008, [6]Hope 2010
Human	Syndromic	Sales	Prescription Medical Sales	[5]Buehler 2008
Human	Syndromic	Sales	Grocery purchase trends	
Human	Syndromic	Sales	Retail data	[67]Bracken 2006
Human	Environmental Syndromic	Social Media News Aggregators	Expert curated accounts Mainstream news	[50]Keller 2009
Human	Environmental Syndromic	Social Media	SMS (Short Message Service)	[18]Freifeld 2010, [68]Gomez 2008, [69]Gow 2010
Human	Environmental Syndromic	Social Media	SMS texting	[70][71]Lewis 2011, [68]Gomez 2009,2010
Human	Environmental Syndromic	Social Media	SMS messaging, microblogging, emailing, social networking, internet chatting, blogging, online news reporting, video/radio reporting, health expert reporting	[50]Keller 2009, [72]Khan 2010 [54]Wilson 2009 [73]Boulos 2011 [74]Lyon 2011
Human	Environmental Syndromic	Social Media	Twitter	[75]Sigorini 2011

Table S1 References

1. Mueller DH, Abeku TA, Okia M, Rapuoda B, Cox J (2009) Costs of early detection systems for epidemic malaria in highland areas of Kenya and Uganda. Malaria Journal 8. Available: ://WOS:000264573500003; http://www.malariajournal.com/content/pdf/1475-2875-8-17.pdf.

2. Polgreen PM, Chen ZQ, Segre AM, Harris ML, Pentella MA, et al. (2009) Optimizing Influenza Sentinel Surveillance at the State Level. Am J Epidemiol 170: 1300–1306. doi:10.1093/aje/kwp270.

3. Scheitle CP (2011) Google's Insights for Search: A Note Evaluating the Use of Search Engine Data in Social Research. Soc Sci Q 92: 285–295. doi:10.1111/j.1540-6237.2011.00768.x.

4. Plagianos MG, Wu WY, McCullough C, Paladini M, Lurio J, et al. (2011) Syndromic surveillance during pandemic (H1N1) 2009 outbreak, New York, New York, USA. Emerg Infect Dis 17: 1724–1726. doi:10.3201/eid1709.101357.

5. Buehler JW, Sonricker A, Paladini M, Soper P, Mostashari F (2008) Syndromic surveillance practice in the United States: findings from a survey of state, territorial, and selected local health departments. Advances in Disease Surveillance 6: 1–20.

6. Hope KG, Merritt TD, Durrheim DN, Massey PD, Kohlhagen JK, et al. (2010) Evaluating the utility of emergency department syndromic surveillance for a regional public health service. Commun Dis Intell 34: 310–318.

7. Tokars JI, English R, McMurray P, Rhodes B (2010) Summary of data reported to CDC's national automated biosurveillance system, 2008. BMC Med Inform Decis Mak 10. Available: ://WOS:000279921700001.

8. Bradley CA, Rolka H, Walker D, Loonsk J (2005) BioSense: implementation of a National Early Event Detection and Situational Awareness System. MMWR Morb Mortal Wkly Rep 54 Suppl: 11–19.

9. Koski E (2011) Clinical Laboratory Data for Biosurveillance; Infectious Disease Informatics and Biosurveillance. In: Castillo-Chavez CC, editor. Integrated Series in Information Systems. Springer US, Vol. 27. pp. 67–87. Available: http://dx.doi.org/10.1007/978-1-4419-6892-0_4.

10. Huaman MA, Araujo-Castillo RV, Soto G, Neyra JM, Quispe JA, et al. (2009) Impact of two interventions on timeliness and data quality of an electronic disease surveillance system in a resource limited setting (Peru): a prospective evaluation. BMC Med Inform Decis Mak 9: 16. doi:10.1186/1472-6947-9-16.

11. Ashford DA, Kaiser RM, Bales ME, Shutt K, Patrawalla A, et al. (2003) Planning against biological terrorism: Lessons from outbreak investigations. Emerging Infectious Diseases 9: 515–519.

12. Thomas-Bachli A, Pearl D, Friendship R, Berke O (2012) Suitability and limitations of portion-specific abattoir data as part of an early warning system for emerging diseases of swine in Ontario. Bmc Veterinary Research 8: 3.

13. Bartlett PC, Van Buren JW, Neterer M, Zhou C (2010) Disease surveillance and referral bias in the veterinary medical database. Prev Vet Med 94: 264–271. doi:10.1016/j.prevetmed.2010.01.007.

14. Shmueli G, Burkom H (2010) Statistical Challenges Facing Early Outbreak Detection in Biosurveillance. Technometrics 52: 39–51. doi:10.1198/tech.2010.06134.

15. Malik MT, Gumel A, Thompson LH, Strome T, Mahmud SM (2011) "Google flu trends" and emergency department triage data predicted the 2009 pandemic H1N1 waves in Manitoba. Can J Public Health 102: 294–297.

16. Rabadan R, Calman N, Hripcsak G (2009) Next Generation Syndromic Surveillance: Molecular Epidemiology, Electronic Health Records and the Pandemic Influenza A (H1N1) Virus. PLoS Curr 1. doi:10.1371/currents.RRN1012.

17. Balabanova Y, Gilsdorf A, Buda S, Burger R, Eckmanns T, et al. (2011) Communicable Diseases Prioritized for Surveillance and Epidemiological Research: Results of a Standardized Prioritization Procedure in Germany, 2011. PLoS One 6. Available: ://WOS:000295963300017.

18. Freifeld CC, Chunara R, Mekaru SR, Chan EH, Kass-Hout T, et al. (2010) Participatory Epidemiology: Use of Mobile Phones for Community-Based Health Reporting. PLos Med 7. Available: ://WOS:000285499600003.

19. Clements AC, Pfeiffer DU (2009) Emerging viral zoonoses: frameworks for spatial and spatiotemporal risk assessment and resource planning. Vet J 182: 21–30. doi:10.1016/j.tvjl.2008.05.010.

20. Keller M, Freifeld CC, Brownstein JS (2009) Automated vocabulary discovery for geo-parsing online epidemic intelligence. BMC Bioinformatics 10. Available: ://WOS:000272335800001.

21. Charland KML, Buckeridge DL, Sturtevant JL, Melton F, Reis BY, et al. (2009) Effect of environmental factors on the spatio-temporal patterns of influenza spread. Epidemiol Infect 137: 1377–1387. doi:10.1017/s0950268809002283.

22. DeGroote JP, Sugumaran R, Brend SM, Tucker BJ, Bartholomay LC (2008) Landscape, demographic, entomological, and climatic associations with human disease incidence of West Nile virus in the state of Iowa, USA. International Journal of Health Geographics 7. Available: ://WOS:000258233300001.

23. Cox J, Abeku TA (2007) Early warning systems for malaria in Africa: from blueprint to practice. Trends in Parasitology 23: 243–246. doi:10.1016/j.pt.2007.03.008.

24. Kristan M, Abeku TA, Beard J, Okia M, Rapuoda B, et al. (2008) Variations in entomological indices in relation to weather patterns and malaria incidence in East African highlands: implications for epidemic prevention and control. Malaria Journal 7. Available: ://WOS:000261438800001; http://www.malariajournal.com/content/pdf/1475-2875-7-231.pdf.

25. Chui KKH, Wenger JB, Cohen SA, Naumova EN (2011) Visual Analytics for Epidemiologists: Understanding the Interactions Between Age, Time, and Disease with Multi-Panel Graphs. PLoS One 6. Available: ://WOS:000287369200002.

26. Baker S, Holt KE, Clements ACA, Karkey A, Arjyal A, et al. (2011) Combined high-resolution genotyping and geospatial analysis reveals modes of endemic urban typhoid fever transmission. Open Biology 1: 110008–110008. doi:10.1098/rsob.110008.

27. Atkinson M, Piskorski J, Pouliquen B, Steinberger R, Tanev H, et al. (2008) Online-monitoring of security-related events Association for Computational Linguistics. pp. 145–148.

28. Berger M, Shiau R, Weintraub JM (2006) Review of syndromic surveillance: implications for waterborne disease detection. J Epidemiol Community Health 60: 543–550. doi:10.1136/jech.2005.038539.

29. Ortiz JR, Zhou H, Shay DK, Neuzil KM, Fowlkes AL, et al. (2011) Monitoring Influenza Activity in the United States: A Comparison of Traditional Surveillance Systems with Google Flu Trends. PloS One 6: e18687. doi:10.1371/journal.pone.0018687.

30. Ginsberg J, Mohebbi MH, Patel RS, Brammer L, Smolinski MS, et al. (2009) Detecting influenza epidemics using search engine query data. Nature 457: 1012–1014. doi:10.1038/nature07634.

31. Hulth A, Rydevik G (2011) GET WELL: an automated surveillance system for gaining new epidemiological knowledge. Bmc Public Health 11. Available: ://WOS:000290848500001.

32. Brownstein JS, Freifeld CC (2008) Evaluation of Internet-Based Informal Surveillance for Global Infectious Disease Intelligence. International Journal of Infectious Diseases 12: E193–E194. doi:10.1016/j.ijid.2008.05.481.

33. Brownstein JS, Freifeld CC, Madoff LC (2009) Digital disease detection--harnessing the Web for public health surveillance. N Engl J Med 360: 2153–2155, 2157. doi:10.1056/NEJMp0900702.

34. Chan T-C, King C-C (2011) Surveillance and Epidemiology of Infectious Diseases using Spatial and Temporal Lustering Methods; Infectious Disease Informatics and

Biosurveillance. In: Castillo-Chavez CC, editor. Integrated Series in Information Systems. Springer US, Vol. 27. pp. 207–234. Available: http://dx.doi.org/10.1007/978-1-4419-6892-0_10.

35. Hulth A, Andersson Y, Hedlund KO, Andersson M (2010) Eye-Opening Approach to Norovirus Surveillance. Emerging Infectious Diseases 16: 1319–1321. doi:10.3201/eid1608.100093.

36. Polgreen PM, Chen YL, Pennock DM, Nelson FD (2008) Using Internet Searches for Influenza Surveillance. Clin Infect Dis 47: 1443–1448. doi:10.1086/593098.

37. Madoff LC, Fisman DN, Kass-Hout T (2011) A New Approach to Monitoring Dengue Activity. Plos Neglected Tropical Diseases 5. Available: ://WOS:000291099100049.

38. McNamara T (2007) The role of zoos in biosurveillance. International Zoo Yearbook 41: 12–15. doi:10.1111/j.1748-1090.2007.00019.x.

39. Dorea FC, Sanchez J, Revie CW (2011) Veterinary syndromic surveillance: Current initiatives and potential for development. Prev Vet Med 101: 1–17. doi:10.1016/j.prevetmed.2011.05.004.

40. Kloeze H, Berezowski J, Bergeron L, de With N, Duizer G, et al. (2011) A Minimum Data Set of Animal Health Laboratory Data to allow for Collation and Analysis across Jurisdictions for the Purpose of Surveillance. Transboundary and Emerging Diseases: no–no. doi:10.1111/j.1865-1682.2011.01264.x.

41. Dubrawski A, Sabhnani M, Knight M, Baysek M, Neill D, et al. (2009) T-Cube Web Interface in support of real-time bio-surveillance program IEEE. pp. 495–495.

42. Hoorfar J, Wagner M, Jordan K, Bouquin SL, Skiby J (2011) Towards biotracing in food chains. International Journal of Food Microbiology 145, Supplement 1: S1–S4. doi:10.1016/j.ijfoodmicro.2010.04.028.

43. Knutsson R, van Rotterdam B, Fach P, De Medici D, Fricker M, et al. (2011) Accidental and deliberate microbiological contamination in the feed and food chains - How biotraceability may improve the response to bioterrorism. Int J Food Microbiol 145: S123–S128. doi:10.1016/j.ijfoodmicro.2010.10.011.

44. Olano JP, Walker DH (2011) Diagnosing Emerging and Reemerging Infectious Diseases The Pivotal Role of the Pathologist. Arch Pathol Lab Med 135: 83–91.

45. Sintchenko V, Gallego B (2009) Laboratory-Guided Detection of Disease Outbreaks Three Generations of Surveillance Systems. Arch Pathol Lab Med 133: 916–925. doi:10.1043/1543-2165-133.6.916.

46. Churchill R, Lorence D, Richards M (2010) Proposed Model for ONCHIT Pre-Case Biosurveillance Using Multiple Array Sensing and Non-Invasive Data Capture. J Med Syst 34: 695–700. doi:10.1007/s10916-009-9283-8.

47. Fuller S (2010) Tracking the Global Express: new tools addressing disease threats across the world. Epidemiology 21: 769–771. doi:10.1097/EDE.0b013e3181f56757.

48. Collier N (2010) What's unusual in online disease outbreak news? J Biomed Semantics 1: 2. doi:10.1186/2041-1480-1-2.

49. Grady N, Vizenor L, Marin J, Peitersen L (2008) Bio-surveillance Event Models, Open Source Intelligence, and the Semantic Web. Biosurveillance and Biosecurity: 22–31. doi:10.1007/978-3-540-89746-0_3.

50. Keller M, Blench M, Tolentino H, Freifeld CC, Mandl KD, et al. (2009) Use of Unstructured Event-Based Reports for Global Infectious Disease Surveillance. Emerg Infect Dis 15: 689–695. doi:10.3201/eid1505.081114.

51. Torii M, Yin LL, Nguyen T, Mazumdar CT, Liu HF, et al. (2011) An exploratory study of a text classification framework for Internet-based surveillance of emerging epidemics. Int J Med Inform 80: 56–66. doi:10.1016/j.ijmedinf.2010.10.015.

52. Piskorski J, Tanev H, Atkinson M, van der Goot E, Zavarella V (2011) Online News Event Extraction for Global Crisis Surveillance; Transactions on Computational Collective Intelligence V. In: Nguyen N, editor. Lecture Notes in Computer Science. Springer Berlin / Heidelberg, Vol. 6910. pp. 182–212. Available: http://dx.doi.org/10.1007/978-3-642-24016-4_10.

53. Tanev H, Piskorski J, Atkinson M (2008) Real-Time News Event Extraction for Global Crisis Monitoring; Natural Language and Information Systems. In: Kapetanios ES, editor. Lecture Notes in Computer Science. Springer Berlin / Heidelberg, Vol. 5039. pp. 207–218. Available: http://dx.doi.org/10.1007/978-3-540-69858-6_21.

54. Wilson K, Brownstein JS (2009) Early detection of disease outbreaks using the Internet. Cmaj 180: 829–831. doi:10.1503/cmaj.090215.

55. Mykhalovskiy E, Weir L (2006) The global public health intelligence network and early warning outbreak detection - A Canadian contribution to global public health. Can J Public Health-Rev Can Sante Publ 97: 42–44.

56. Dragu N, Elkhoury F, Miyazaki T, Morelli RA, di Tada N (2010) Ontology-Based Text Mining for Predicting Disease Outbreaks.

57. Pultorak E, Nadler Y, Travis D, Glaser A, McNamara T, et al. (2011) Zoological institution participation in a West Nile Virus surveillance system: implications for public health. Public Health 125: 592–599. doi:10.1016/j.puhe.2011.03.013.

58. Adler PH, Tuten HC, Nelder MP (2011) Arthropods of Medicoveterinary Importance in Zoos. Annual Review of Entomology, Vol 56 56: 123–142. doi:10.1146/annurev-ento-120709-144741.

59. Baer A, Duchin J (2009) Monitoring Staphylococcus Infection Trends with Biosurveillance Data. Available: http://isdsjournal.org/articles/3205.pdf.

60. Trifonov V, Khiabanian H, Rabadan R (2009) Geographic Dependence, Surveillance, and Origins of the 2009 Influenza A (H1N1) Virus. New England Journal of Medicine 361: 115–119. doi:10.1056/NEJMp0904572.

61. Babin SM, Burkom HS, Holtry RS, Tabernero NR, Davies-Cole JO (2008) Air Quality Effects on Health-Indicator Data in Disease Outbreak Surveillance. Johns Hopkins Apl Technical Digest 27: 393–402.

62. Ortiz-Pelaez A, Pfeiffer DU, Tempia S, Otieno FT, Aden HH, et al. (2010) Risk mapping of Rinderpest sero-prevalence in Central and Southern Somalia based on spatial and network risk factors. BMC Vet Res 6. Available: ://WOS:000278259600001.

63. Vernon MC, Keeling MJ (2009) Representing the UK's cattle herd as static and dynamic networks. Proc R Soc B-Biol Sci 276: 469–476. doi:10.1098/rspb.2008.1009.

64. Briand S, Mounts A, Chamberland M (2011) Challenges of global surveillance during an influenza pandemic. Public Health 125: 247–256. doi:10.1016/j.puhe.2010.12.007.

65. Polgreen PM, Nelson FD, Neumann GR, Weinstein RA (2007) Use of Prediction Markets to Forecast Infectious Disease Activity. Clinical Infectious Diseases 44: 272–279. doi:10.1086/510427.

66. Pfeiffer T, Almenberg J (2010) Prediction markets and their potential role in biomedical research--a review. Biosystems 102: 71–76. doi:10.1016/j.biosystems.2010.09.005.

67. Bracken T (2006) Early Warning: The Role of Retail Data Biosurveillance in our Country's Preparedness Efforts. Available: http://www.BioSentinel.net.

68. Gomez A (2008) Connecting communities of need with public health: can SMS text-messaging improve outreach communication? 2008 41st Annual Hawaii International Conference on System Sciences: 1043–10521052.

69. Gow GA, Waidyanatha N, Mary VP (2010) Using Mobile Phones in a Real-time Biosurveillance Program: Lessons from the frontlines in Sri Lanka and India. In: Michael K, editor. Proceedings of the 2010 Ieee International Symposium on Technology and Society: Social Implications of Emerging Technologies. IEEE International Symposium on Technology and Society. New York: Ieee. pp. 366–374. Available: ://WOS:000287498800040.

70. Lewis SH, Chretien JP (2008) The Potential Utility of Electronic Disease Surveillance Systems in Resource-Poor Settings. Johns Hopkins Apl Technical Digest 27. Available: www.jhuapl.edu/techdigest/TD/td2704/happellewis.pdf.

71. Lewis SH, Holtry RS, Loschen WA, Wojcik R, Hung L, et al. (2011) The Collaborative Experience of Creating the National Capital Region Disease Surveillance Network. J Public Health Manag Pract 17: 248–254. doi:10.1097/PHH.0b013e3181f9eeda.

72. Khan AS, Fleischauer A, Casani J, Groseclose SL (2010) The Next Public Health Revolution: Public Health Information Fusion and Social Networks. Am J Public Health 100: 1237–1242. doi:10.2105/ajph.2009.180489.

73. Boulos KMN, Resch B, Crowley DN, Breslin JG, Sohn G, et al. (2011) Crowdsourcing, citizen sensing and sensor web technologies for public and environmental health surveillance and crisis management: trends, OGC standards and application examples. Int J Health Geogr 10: 67. doi:10.1186/1476-072x-10-67.

74. Lyon A, Nunn M, Grossel G, Burgman M (2011) Comparison of Web-Based Biosecurity Intelligence Systems: BioCaster, EpiSPIDER and HealthMap. Transboundary and Emerging Diseases: no–no. doi:10.1111/j.1865-1682.2011.01258.x.

75. Gulli A, Signorini A (2005) The indexable web is more than 11.5 billion pages 1062789: ACM. pp. 902–903. doi:10.1145/1062745.1062789.
